# Temporal trends in mortality and provision of intensive care in younger women and men with acute myocardial infarction or stroke

**DOI:** 10.1186/s13054-022-04299-0

**Published:** 2023-01-12

**Authors:** Ketina Arslani, Janna Tontsch, Atanas Todorov, Bianca Gysi, Mark Kaufmann, Fabian Kaufmann, Alexa Hollinger, Karin Wildi, Hamid Merdji, Julie Helms, Martin Siegemund, Catherine Gebhard, Caroline E. Gebhard, on behalf of the Swiss Society of Intensive Care Medicine

**Affiliations:** 1 Department of Cardiology, University Hospital Basel, Basel, Switzerland; 2 Department of Cardiology, Rigshospitalet, Copenhagen University Hospital, Copenhagen, Denmark; 3 Intensive Care Unit, Department of Acute Medicine, University Hospital Basel, Petersgraben 4, 4031 Basel, Switzerland; 4 Department of Anesthesiology, University Hospital Basel, Basel, Switzerland; 5 Department of Nuclear Medicine, University Hospital Zurich, Zurich, Switzerland; 6 Center for Molecular Cardiology, University of Zurich, Zurich, Switzerland; 7 University of Basel, Basel, Switzerland; 8 Critical Care Research Group, The University of Queensland, Brisbane, Australia; 9 Cardiovascular Research Group, Basel, Switzerland; 10 Université de Strasbourg (UNISTRA), Faculté de Médecine; Hôpitaux universitaires de Strasbourg, Service de Médecine Intensive-Réanimation, Nouvel Hôpital Civil, Strasbourg, France; 11 INSERM (French National Institute of Health and Medical Research), UMR 1260, Regenerative Nanomedicine (RNM), FMTS, Strasbourg, France; 12 Department of Cardiology, University Hospital Bern, Bern, Switzerland

**Keywords:** Gender gap, Gender bias, Mortality trend, Critical care, Cardiovascular diseases, Sex disparities

## Abstract

**Background:**

Timely management of acute myocardial infarction (AMI) and acute stroke has undergone impressive progress during the last decade. However, it is currently unknown whether both sexes have profited equally from improved strategies. We sought to analyze sex-specific temporal trends in intensive care unit (ICU) admission and mortality in younger patients presenting with AMI or stroke in Switzerland.

**Methods:**

Retrospective analysis of temporal trends in 16,954 younger patients aged 18 to ≤ 52 years with AMI or acute stroke admitted to Swiss ICUs between 01/2008 and 12/2019.

**Results:**

Over a period of 12 years, ICU admissions for AMI decreased more in women than in men (− 6.4% in women versus − 4.5% in men, *p* < 0.001), while ICU mortality for AMI significantly increased in women (OR 1.2 [1.10–1.30], *p* = 0.032), but remained unchanged in men (OR 0.99 [0.94–1.03], *p* = 0.71). In stroke patients, ICU admission rates increased between 3.6 and 4.1% per year in both sexes, while ICU mortality tended to decrease only in women (OR 0.91 [0.85–0.95, *p* = 0.057], but remained essentially unaltered in men (OR 0.99 [0.94–1.03], *p* = 0.75). Interventions aimed at restoring tissue perfusion were more often performed in men with AMI, while no sex difference was noted in neurovascular interventions.

**Conclusion:**

Sex and gender disparities in disease management and outcomes persist in the era of modern interventional neurology and cardiology with opposite trends observed in younger stroke and AMI patients admitted to intensive care. Although our study has several limitations, our data suggest that management and selection criteria for ICU admission, particularly in younger women with AMI, should be carefully reassessed.

**Graphical Abstract:**

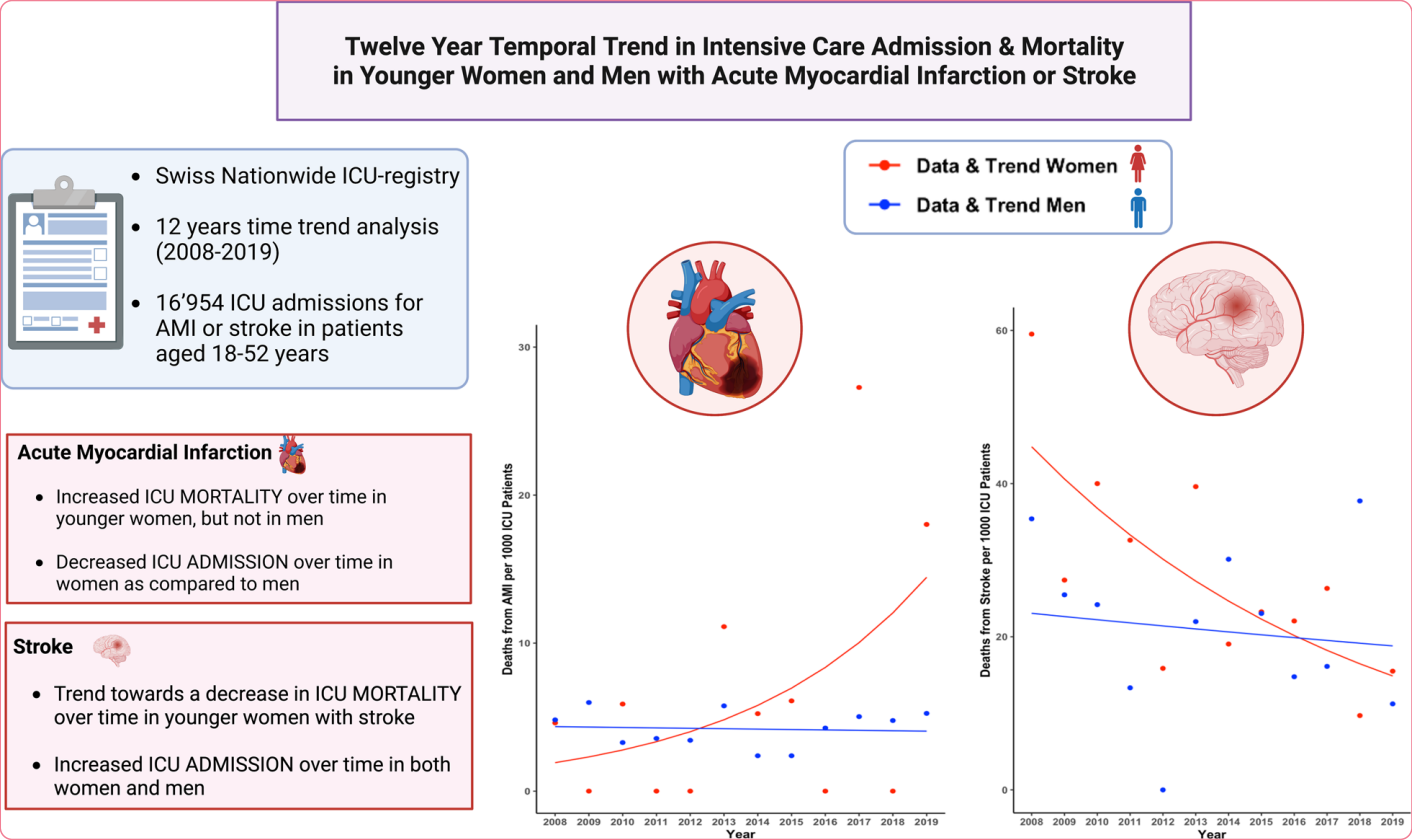

**Supplementary Information:**

The online version contains supplementary material available at 10.1186/s13054-022-04299-0.

## Background

Despite major therapeutic advances during the last decade, cardiovascular and cerebrovascular diseases are still among the leading causes of death and serious long-term disability worldwide [1, 2]. While overall incidence and case fatality rates for stroke or acute myocardial infarction (AMI) have steadily declined since the 1980s owing to improved primary and secondary prevention and technical refinement [3, 4], concerns about different trends in diagnostics, treatment and outcome in women and men still remain. In fact, while overall stroke incidence has decreased over the last decade [3, 5–7], recent studies report increasing stroke incidence and case fatality rates in younger individuals [8–10] with inconsistent data being reported about gender differences in terms of stroke interventions, outcome and care [7, 11, 12].

A rising incidence and case fatality for AMI have been observed in younger women, but not in men [13–15]. Moreover, in younger men, the incidence rate of hospitalization and excess mortality has even decreased over time [4, 16]. Although exact mechanisms accounting for these differential time trends in younger women and men are lacking, an increase in comorbidities and cardiovascular risk factors, in particular smoking, gender differences in clinical presentation and, thus, treatment delays have been proposed to account for this finding [14, 17, 18]. Besides differences in therapeutic options and shifts in the distribution of traditional and non-traditional risk factors over time, gender inequality in the provision of resources and structural bias may impact cardio- and neurovascular disease outcomes in the younger population [19]. We therefore sought to analyze gender-specific temporal trends in intensive care unit (ICU) admission and mortality over a period of 12 years (2008–2019) in younger patients aged 18–52 years presenting with AMI or ischemic stroke to Swiss hospitals.

## Methods

Data were obtained from the Swiss ICU-registry (MDSi - Minimal Dataset for ICUs) of the Swiss Society of Intensive Care Medicine (SSICM), which contains data from all 86 certified ICUs in Switzerland as previously described [19, 20]. ICU admissions and mortality for patients with AMI or ischemic stroke were obtained from the MDSi database. Data of all patients identified with AMI or ischemic stroke in all Swiss hospitals were provided by the Swiss Federal Statistical Office (FSO). The Ethics committee of Northwestern Switzerland approved this procedure (EKNZ UBE-15/47). The study was carried out according to the principles of the Declaration of Helsinki of 1975. We adhered to the STROBE reporting guidelines for observational studies (Additional file 1: Table S1) [21].

### Variables and outcomes

The primary outcome measure of this study was the temporal change in ICU mortality over 12 years. Secondary outcome measures included ICU admission rates and disease severity over time. From the MDSi dataset, we extracted demographic data including sex, age, year of admission, information about residence prior to ICU admission, admission diagnosis, therapeutic (including invasive) interventions before ICU admission, the Simplified Acute Physiology Score (SAPS II) [22], the Nine Equivalents of Nursing Manpower Use Score (NEMS) [23] of the first nursing shift and average NEMS per shift, as well as ICU mortality for patients who were admitted for AMI or stroke between 2008 and 2019 to an ICU in Switzerland. The selection of ischemic stroke patients was made based on the overlapping pathogenesis and risk factors of AMI and ischemic stroke and the provision of a large, uniform patient population. The SAPS II score estimates disease severity and, thus, mortality in patients admitted to the ICU. For score calculation, the worst physiological values as indicators of organ function, age, type of admission and information on previous health status (chronic diseases such as cancer, metastatic carcinoma, or hematologic malignancies) are collected within the first 24 h of ICU admission. While the SAPS II score reflects the situation at ICU admission (first 24 h), the NEMS parameters are recorded daily (per shift) until ICU discharge. Assessments include the daily amount of organ support measures (vasoactive medications, mechanical ventilation and breathing aids, renal replacement therapy), interventions during ICU stay inside (e.g., endotracheal intubation, placement of a pacemaker, cardioversion, endoscopy) and outside the ICU (e.g., surgical intervention or diagnostic procedures).

From the FSO, mortality data and diagnoses of the same period were obtained as for the MDSi dataset. Overall mortality was defined as in-hospital deaths of all patients admitted to Swiss hospitals. Younger age was defined as age ≤ 52 years. This age cutoff was based on the average menopausal age for women in Switzerland [24] to form a premenopausal female cohort and on previous studies with younger age ranging from ≤ 44 to 55 years [9–14, 25]. ICU mortality was defined as all-cause mortality during ICU stay.

### Statistical analysis

Data from the MDSi database were filtered for primary admission diagnosis of AMI or ischemic stroke and patients aged > 18 and ≤ 52 years. For the numbers of total admissions and deaths, Poisson models were built for primary trend analysis, which included sex and year of admission as covariates. Data from the FSO on death numbers for the respective diagnosis per sex and age > 18 and ≤ 52 years were analyzed in parallel as surrogates of overall mortality development. Analysis of mortality (deaths per admission) was performed with a logistic model including all possible covariates from the SGI data set. Variable reduction on the logistic model was performed both stepwise and using a penalized regression (LASSO). Both reduction strategies resulted in selection of SAPS and NEMS scores as the most important explanatory variables. For both explanatory variables, the receiver operating characteristic (ROC) for death prediction was analyzed, and the best predictive score as defined by Youden's index [26] was analyzed for yearly trend. Baseline characteristics of the ICU population were compared between men and women using t-test, parametric rank sum test, or Chi-square test as appropriate. Statistical analysis was performed using R version 3.6.1 with the packages glmnet, ROCit, and quantreg [27].

## Results

From January 2008 to December 2019, a total of 292,103 individuals were diagnosed with either an acute stroke (n = 215,397) or an AMI (n = 270,768) in Switzerland. Among individuals aged > 18 and ≤ 52 years, 34,024 were diagnosed with AMI (5293 women, 15.6%) and 18,043 (6774 women, 37.5%) with stroke.

### Baseline characteristics, ICU admission and mortality temporal trends in ICU patients with AMI

Over the 12-year period, 13´449 [16.6% women] individuals > 18 and ≤ 52 years were admitted to an ICU for AMI. No significant age difference was observed between women and men (45 ± 6 vs 45 ± 5 years old, *p* = 0.229). There was no significant sex difference in SAPS II, while the initial NEMS after admission (17.8 ± 8.0 vs 17.1 ± 7.7 points, *p* < 0.001) and the average NEMS per shift (16.8 ± 6.4 vs 16.3 ± 6.6 points, *p* < 0.001) were higher in men than in women. Lengths of ICU stay (LOS) and the absolute number of deaths over the whole period were similar between women and men. Prior to ICU admission, men with AMI underwent more frequently cardiovascular interventions (percutaneous coronary intervention [PCI]: 6222 [55.5%] in men vs 1081 [48.5%] in women, *p* < 0.001; CABG surgery: 987 [8.8%] in men vs 106 [4.75%] in women, *p* < 0.001). In general, men received more subsequent interventions during ICU stay (intubation, cardioversion, endoscopies) (622[5.54%] in men vs 148 [6.63%] in women, *p* = 0.049), more organ support such as mechanical ventilation (1330 [11.9%] in men vs 185 [8.3%] in women, *p* < 0.001) and vasoactive support (2520 [22.5%] in men vs 437 [19.6%] in women, *p* = 0.003). Table 1 lists the baseline characteristics of the ICU study population.

**Table 1:** Baseline characteristics of the ICU study populations aged ≤ 52 years stratified by sex

	Acute myocardial infarction age ≤ 52 years				Stroke age ≤ 52 years			
	Overall n = 13,449	Men n = 11,218 (83.4%)	Women n = 2231 (16.6%)	*p*-value	Overall n = 3505	Men n = 2116 (60.4%)	Women n = 1389 (39.6%)	*p*-value
Age (years), mean ± SD	45 ± 5.4	45 ± 5.4	45 ± 5.7	0.229	43 ± 7.9	44 ± 7.3	41 ± 8.5	< 0.001
Transfer from other hospital, n (%)	3140 (23.3)	2646 (23.6)	494 (22.1)	0.176	482 (13.8)	277 (13.1)	205 (14.8)	0.353
Direct admissions, n (%)	9684 (72.0)	8042 (71.7)	1642 (73.6)		2591 (73.9)	157 74.3)	1018 (73.3)	
Other, n (%)	625 (4.6)	530 (4.7)	95 (4.3)		432 (12.3)	266 (12.6)	166 (12.0)	
SAPS II (points) - mean ± SD	18.37 ± 9.32	18.34 ± 9.26	18.53 ± 9.61	0.382	20.16 ± 12.25	20.47 ± 12.47	19.69 ± 11.9	0.061
NEMS score (points)								
1st shift, mean ± SD	17.68 ± 7.93	17.80 ± 8.00	17.05 ± 7.56	< 0.001	17.72 ± 8.92	17.8 ± 8.98	17.61 ± 8.82	0.542
Average NEMS/shift, mean ± SD	16.72 ± 6.44	16.81 ± 6.40	16.3 ± 6.58	< 0.001	17.91 ± 7.92	18 ± 7.43	17.78 ± 8.61	0.436
LOS (days), median (IQR)	0.93 (0.66–1.44)	0.94 (0.67–1.45)	0.9 (0.61–1.42)	0.273	1.16 (0.86–2.06)	1.16 (0.87–2.09)	1.18 (0.84–2.01)	0.624
Deaths, n (%)	62 (0.46)	50 (0.45)	12 (0.54)	0.678	77 (2.2)	44 (2.08)	33 (2.38)	0.640
Interventions before ICU admission				< 0.001				0.485
PCI, n (%)	7303 (54.3)	6222 (55.46)	1081 (48.45)		31 (0.88)	20 (0.95)	11 (0.79)	
Neurovascular interventions, n (%)	9 (0.07)	8 (0.07)	1 (0.04)		511 (14.58)	312 (14.74)	199 (14.33)	
CABG surgery, n (%)	1093 (8.13)	987 (8.8)	106 (4.75)		1 (0.03)	1 (0.05)	0 (0)	
Heart valve surgery, n (%)	26 (0.19)	18 (0.16)	8 (0.36)		1 (0.03)	1 (0.05)	0 (0)	
Aortic surgery, n (%)	2 (0.01)	1 (0.01)	1 (0.04)		1 (0.03)	1 (0.05)	0 (0)	
Cardiovascular unspecified, n (%)	209 (1.55)	173 (1.54)	36 (1.61)		100 (2.85)	52 (2.46)	48 (3.46)	
Other, n (%)	279 (2.07)	226 (2.01)	53 (2.38)		389 (11.1)	230 (10.87)	159 (11.45)	
None, n (%)	4485 (33.35)	3556 (31.7)	929 (41.64)		2452 (69.96)	1492 (70.51)	960 (69.11)	
Interventions during ICU stay								
Interventions performed outside the ICU, n (%)*	3383 (25.15)	2838 (25.3)	545 (24.43)	0.402	1454 (41.48)	899 (42.49)	555 (39.96)	0.147
Interventions performed inside the ICU, n (%)**	770 (5.73)	622 (5.54)	148 (6.63)	0.049	769 (21.94)	461 (21.79)	308 (22.17)	0.818
Mechanical ventilatory support, n (%)	1515 (11.26)	1330 (11.86)	185 (8.29)	< 0.001	673 (19.2)	425 (20.09)	248 (17.85)	0.111
Supplementary ventilatory care***, n (%)	8862 (65.89)	7483 (66.71)	1379 (61.81)	< 0.001	1710 (48.79)	1078 (50.95)	632 (45.5)	0.002
Vasoactive agents								
One, n (%)	2957 (21.99)	2520 (22.46)	437 (19.59)	0.003	990 (28.25)	595 (28.12)	395 (28.44)	0.868
Multiple, n (%)	684 (5.09)	594 (5.3)	90 (4.03)	0.015	233 (6.65)	144 (6.81)	89 (6.41)	0.694
Renal replacement therapy, n (%)	119 (0.88)	94 (0.84)	25 (1.12)	0.239	13 (0.37)	8 (0.38)	5 (0.36)	1

*AMI*, acute myocardial infarction; *CABG*, coronary artery bypass graft; *LOS*, length of stay in ICU; *PCI*, percutaneous coronary intervention; *SAPS II*, Simplified Acute Physiology Score II; *NEMS*, Nine Equivalents of Nursing Manpower Use Score; *ICU*, intensive care unit. *p*-values are reported for comparison between women and men. Comparisons were performed using t-test, nonparametric rank-sum test, or Chi-square test as appropriate

*Specific interventions outside the ICU: surgical intervention or diagnostic procedures; the intervention/procedure is related to the severity of illness of the patient and makes an extra demand upon manpower efforts in the ICU

**Specific interventions in the ICU: such as endotracheal intubation, introduction of pacemaker, cardioversion, endoscopy

***Spontaneous breathing via endotracheal or tracheostomy, supplementary oxygen with spontaneous breathing

**Fig. 1:**
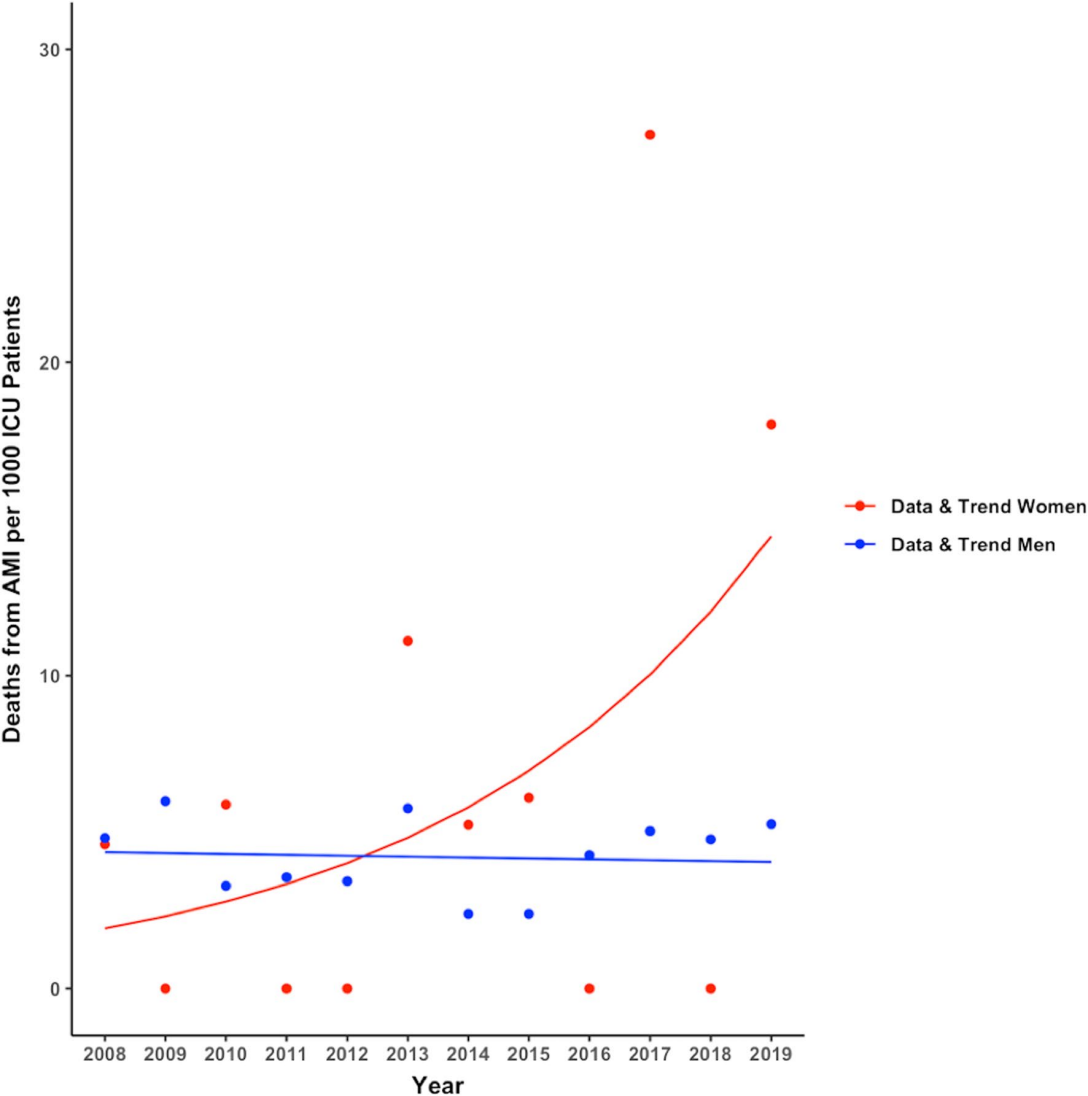
Mortality trends over time in women and men with acute myocardial infarction admitted to intensive care units in Switzerland. Data are presented as observed deaths per 1000 patients in ICU each year (points) and modeled trends (lines)

**Fig. 2:**
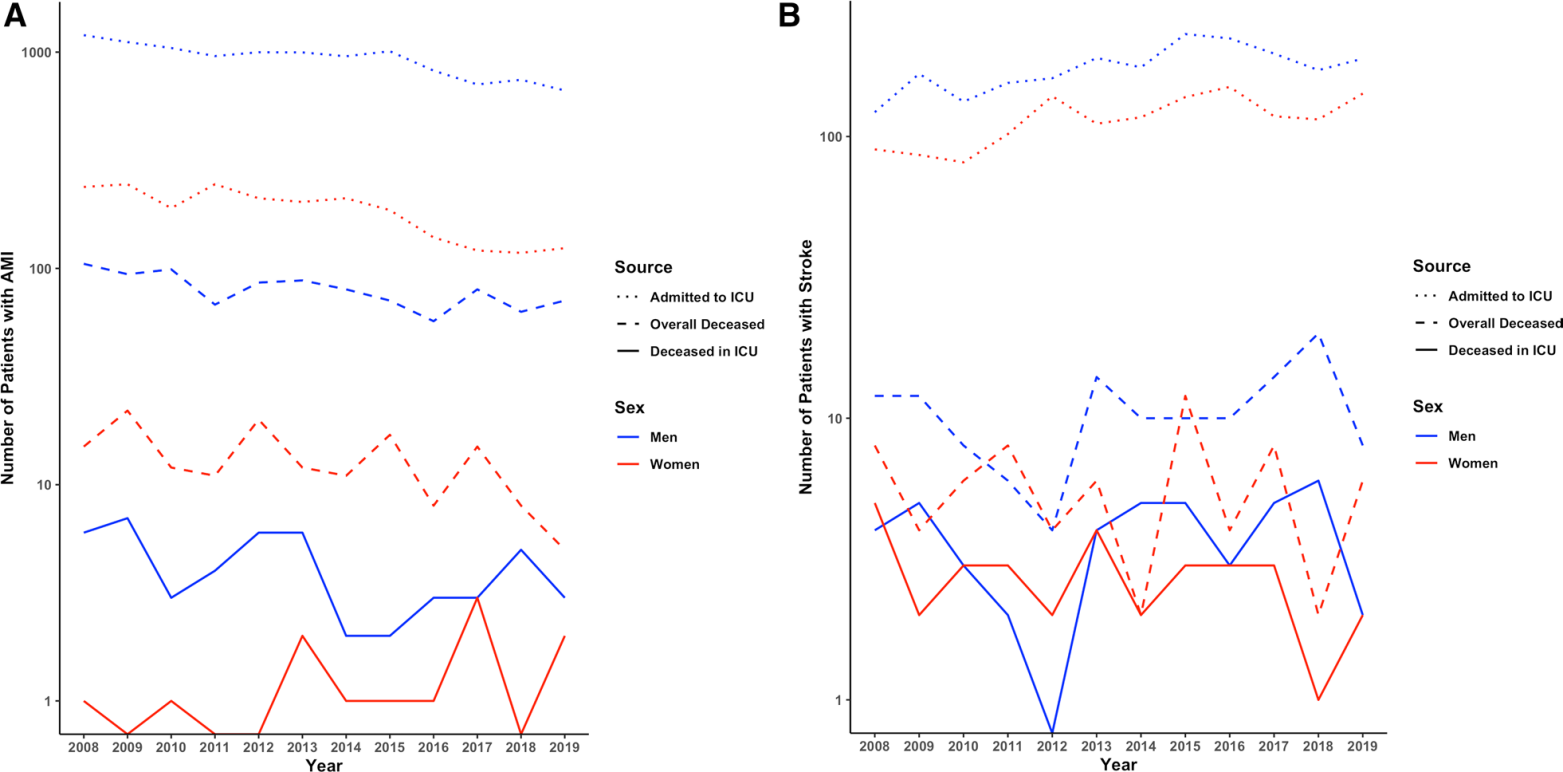
**A** Time trends of overall mortality, ICU mortality, and ICU admission in patients with acute myocardial infarction. **B** Time trends of overall mortality (no statistically significant change over time), ICU mortality (no statistically significant change over time, but tendency toward decline in women), and ICU admission in patients with ischemic stroke. Data are presented as observed numbers of patients in each year, with dotted lines representing overall patients admitted to the ICU, dashed lines showing overall deaths reported to the FSO, and continuous lines showing deaths reported in the ICU. FSO, Swiss Federal Statistical Office; ICU, intensive care unit

**Fig. 3:**
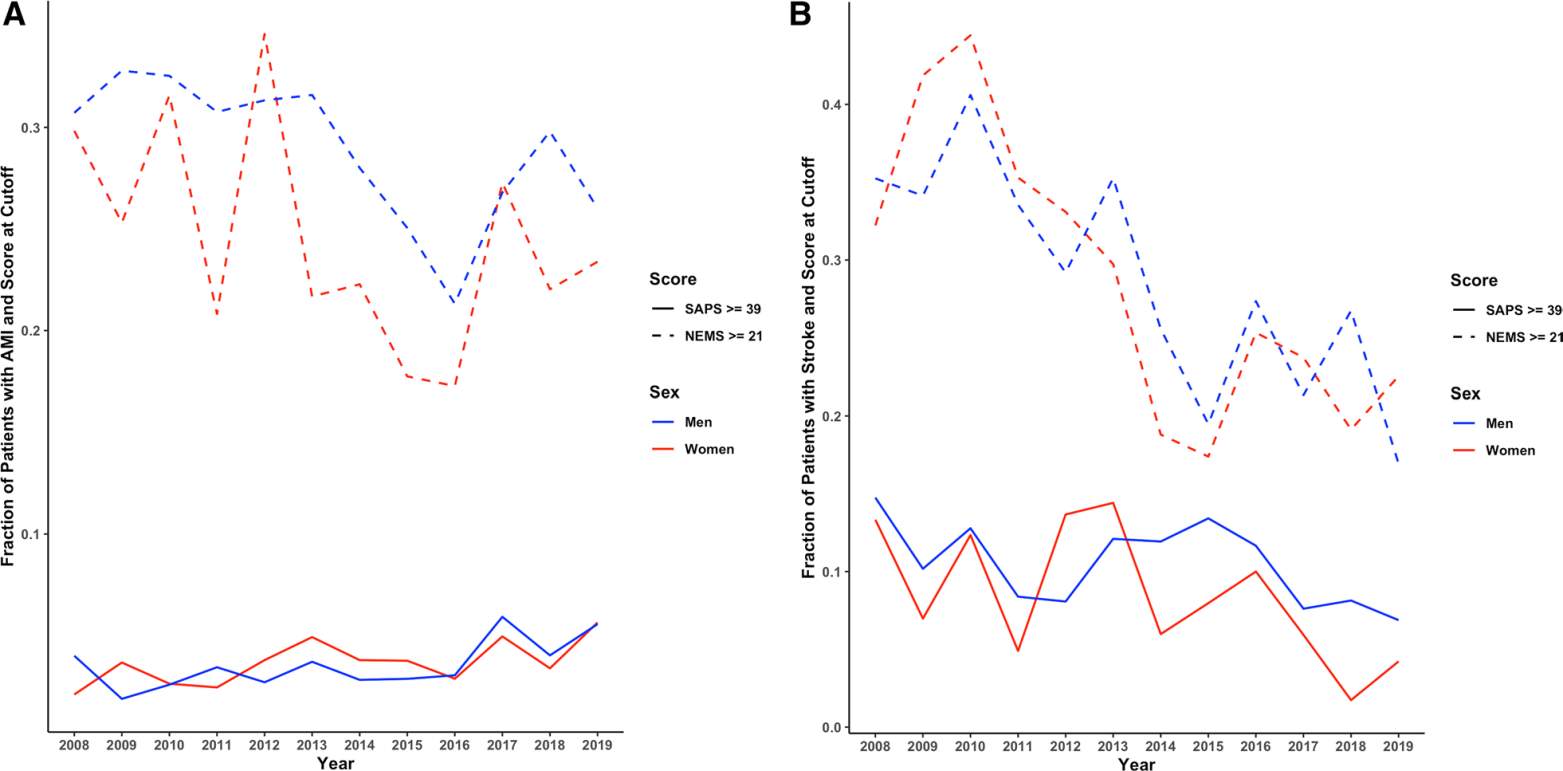
**A** Time trends of disease severity estimated by SAPS II and nursing workload estimated by NEMS in ICU patients admitted with acute myocardial infarction. **B** Time trends of disease severity estimated by SAPS II and nursing workload estimated by NEMS in ICU patients admitted with ischemic stroke. ICU, intensive care unit; NEMS, Nine Equivalents of Nursing Manpower Use Score; SAPS II, Simplified Acute Physiology Score II. Data are presented as fraction of ICU patients at or above the threshold for the respective score (SAPS ≥ 39 or NEMS ≥ 21)

In Switzerland, the overall mortality rate for AMI decreased by − 3.8 to − 6.3% per year between 2008 and 2019 in both sexes**.** Conversely, ICU mortality rates in younger women with AMI, but not in age-matched men, have increased over time (women: OR 1.2 [1.10–1.30], *p* = 0.032; men: (OR 0.99 [0.94–1.03], *p* = 0.71, Fig. 1). Contemporaneously, ICU admission rates for younger women have declined by − 6.4% per year (−[5.8–7% per year], *p* < 0.001) but significantly less so in men (− 4.5% [4.2–4.8%], *p* < 0.001; *p* = 0.002 for women vs men, Fig. 2A). Disease severity as indicated by SAPS II in patients with AMI admitted to the ICU has increased over time (OR 1.05 [1.02–1.08], *p* < 0.001) in both sexes, while NEMS, an indicator of nursing workload, has declined (OR 0.96 [0.95–0.97], *p* = 0.007, Fig. 3A**).** When ICU mortality trends over time were adjusted for SAPS II and NEMS, mortality changes were no longer evident.

### Baseline characteristics, ICU admission and mortality temporal trends in ICU patients with acute stroke

From 2008 to 2019, 3505 [39.6% women] patients aged > 18 and ≤ 52 years were admitted to ICU for stroke. Women with stroke were younger as compared to men (41 ± 9 vs 44 ± 7 years old, *p* < 0.001). No sex differences in NEMS and SAPS II were observed in stroke patients directly after admission (17.8 ± 9.0 points in men vs 17.6 ± 8.8 points in women, *p* = 0.542) and during ICU stay. No significant differences in the LOS and the absolute number of deaths over the whole period were observed between women and men. No sex differences before ICU admission were observed regarding neurovascular interventions in patients with stroke (312 [14.7%] in men vs 199 [14.3%] in women, *p* = 0.485). Interventions during ICU stay (endoscopy, intubation, cardioversion, diagnostic procedures, or subsequent surgeries) were similar in men and women as were organ support measures including mechanical ventilation (425 [20.1%] in men vs 248 [17.9%] in women, *p* = 0.111) and vasoactive support (595 [28.1%] in men vs 395 [28.4%] in women, *p* = 0.868). Noninvasive respiratory support was more frequently used in men (1078 [51.0%] vs 632 [45.5%] in women, *p* = 0.002). Table 1 depicts the baseline characteristics of the ICU study population.

**Fig. 4:**
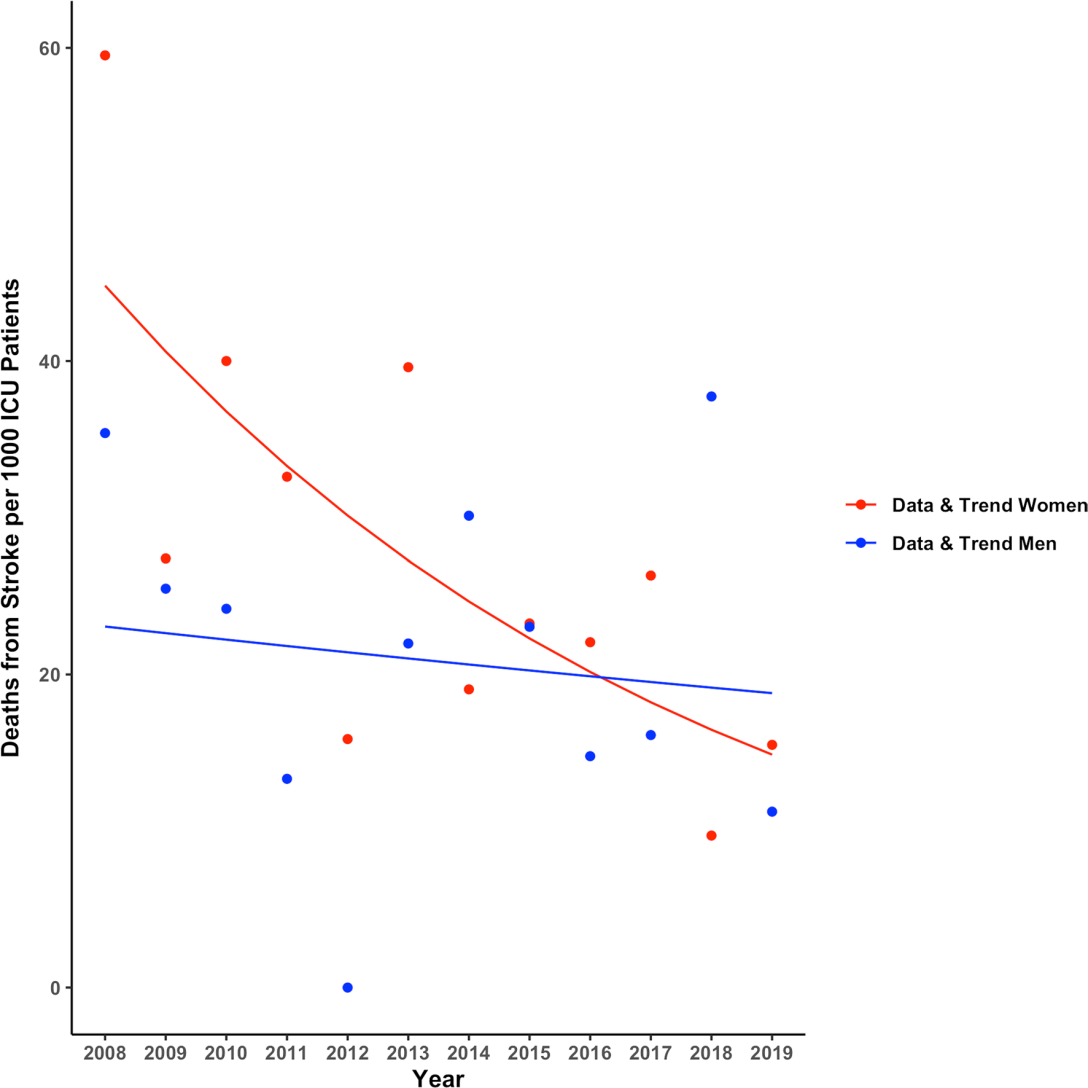
Mortality trend over time in patients with ischemic stroke admitted to intensive care units in Switzerland. Data are presented as observed deaths per 1000 patients in ICU each year (points) and modeled trends (lines)

Overall mortality rates for stroke in Switzerland did not show a significant time trend between 2008 and 2019 in either sex. ICU mortality rates in patients with stroke showed a trend toward a yearly decline of − 4.1% in women (OR 0.91 [0.85–0.95], *p* = 0.057), but remained unchanged in men (OR 0.99 [0.94–1.03] *p* = 0.75, Fig. 4). ICU admission rates for stroke increased over the study period in both sexes by + 3.6 to + 4.1% per year ([2.9–4.2%], *p* < 0.001 in men and [3.3–4.9%], *p* < 0.001 in women, *p* = 0.6 for men vs women, Fig. 2B). Disease severity as indicated by SAPS II in patients with stroke admitted to the ICU decreased over time in women only (women: OR 0.91 [0.89–0.94], *p* = 0.002; men OR 0.96 [0.94–0.98], *p* = 0.08; *p* = 0.067 for women vs men), while nursing workload (NEMS) decreased in both sexes (overall: OR 0.91, *p* < 0.001; women: 0.91 [0.89–0.93], *p* < 0.001; men: 0.92 [0.91–0.93], *p* < 0.001, Fig. 3B). When mortality trends for stroke over time were adjusted for SAPS II and NEMS, mortality changes were no longer evident.

## Discussion

In this large, nationwide registry in Switzerland, sex- and age-specific temporal trends in ICU admission and mortality rates were evaluated in younger patients with AMI or stroke between 2008 and 2019. While we found that the overall mortality rate for AMI in Switzerland has substantially declined by approximately 5% per year over the last 12 years in both men and women, we observed an increasing ICU mortality in younger women with AMI as compared to men. On the other hand, ICU admission rates for AMI decreased significantly more in younger women as compared to men. In stroke patients, a trend toward a decrease in ICU mortality in younger women, but not in men was observed while ICU admission rates increased in both sexes.

The overall decrease in mortality trends observed in women and men with AMI in Switzerland is consistent with published literature and can mainly be attributed to technical refinements, improved therapies, and application of guideline-directed therapies to both sexes leading to a narrowing of the gender gap, which was present in earlier studies [28, 29]. Despite increasing awareness of sex and gender differences in cardiovascular disease manifestation [30, 31], treatment strategies and care [32, 33], younger women still encounter poorer outcomes following an AMI as compared to men [13]. Our data confirm this observation by demonstrating an increase in ICU AMI-related mortality in women ≤ 52 years between 2008 and 2019. Female sex hormones have been discussed to have a protective effect on cardiovascular diseases [34]. Given that the age cutoff in our study population was specifically selected to determine a premenopausal female cohort [24], other factors must outweigh the potential benefit of hormonal cardiovascular protection in critically ill younger women with AMI admitted to ICU. An increase in comorbidities, non-traditional risk factors, and a growing burden of psychosocial stress in younger women, all of them associated with worse cardiovascular outcomes, might account for this time alarming temporal trend [34–36]. However, while ICU mortality increased in younger women, we also show that women’s ICU admission rates declined significantly more over time compared to men despite a similar increase in disease severity in both sexes. Therefore, our data indicate that gender inequalities in access to specialized medicine may impose an additional burden on younger women and that triage decisions for ICU admission might have been applied more strictly to the young female population. Gender differences in disease pathophysiology as symptom perception or presentation and diagnostic biases may contribute to the inequality in ICU admission observed between younger women and men [19]. In our study, a drop in ICU admissions for AMI in 2015 can be noted, which was more pronounced in women. At that time, the use of high-sensitive troponin (hs-cTnT) was implemented in triage decisions, allowing to identify patients with smaller myocardial damage at an earlier stage leading to earlier reperfusion therapy and reduced use of ICU resources [37]. Despite international recommendations for sex-specific hs-cTnT cutoff values with younger women having the lowest thresholds [38], the lack to implement them in clinical practice is still evident [39], potentially leading to underestimation or even misdiagnosis of AMI in younger women [40, 41].

The fact that the increased ICU mortality trend in relation to severity of illness in our study was no longer evident indicates that this trend is explainable by the admission of more severely ill patients over the years—which particularly affects the female population given their higher ICU mortality observed. Consistent with published literature, men with AMI in our study were also more frequently referred to reperfusion therapies and received more often organ support upon ICU admission and during ICU stay than women, including mechanical ventilation and vasoactive medication [34, 42]. This phenomenon is known as the ‘Yentl Syndrome’ [43] and might be particularly pronounced in younger women [44].

In contrast to the decline in overall mortality from AMI in Switzerland, stroke-related mortality has not changed over time. However, an increase in ICU admission rates over time in both sexes, alongside a stronger decline in ICU case severity and ICU mortality in younger women, but not in men, was observed in these patients.

The number of stroke patients, in particular younger demographic groups have multiple risk factors and comorbidities such as coagulopathies, smoking, and recreational drug use, has substantially increased during the last decade [45, 46]. The latter might account for the fact that overall stroke-related mortality rates remained stable over time, despite refinements in treatment strategies such as advanced imaging techniques and invasive reperfusion strategies. While ICU admission is frequently limited to the severely affected patient with the need for more invasive monitoring, advanced organ support measures and management of stroke complications, [47–49] the increase in ICU admissions over time might also mirror such advances in treatment strategies which necessitates more intense observation following, for example, endovascular treatments. In contrast to AMI patients, the temporal alterations in ICU admissions did not differ between men and women, suggesting that the gender gap, still observed in AMI patients, has narrowed in stroke patients. This finding is supported by previous studies, reporting the disappearance of gender disparities in stroke outcomes when access to specialized therapies, such as thrombolysis or stroke unit admission was provided equally for women and men [51–53]. Consistent with this notion, no gender difference was observed in the number of stroke patients being referred for neurovascular interventions, whereas recent studies have even described higher rates of endovascular thrombectomy in women [50, 51]. Less aggressive treatment for stroke prevention in women with atrial fibrillation leading to thromboembolic stroke has been previously discussed as a possible explanation for the higher rates of invasive stroke therapy in women [52]. Given the younger age of our cohort and the lower rates of atrial fibrillation, this may explain the equal rate of interventions observed in our study.

The fact that ICU mortality in our study declined more substantially in female stroke patients than in male patients during the last decade suggests that younger women with stroke have greatly benefited from a closing gender gap in the provision of neurological interventions and intensive care.

There are several limitations to this study that should be pointed out. First, our study is observational and does not provide information on underlying mechanisms. Second, the datasets used contain a limited set of variables. Information on patient demographics, symptoms at presentation, preexisting conditions, cardiovascular risk factors as well as admission criteria or biomarkers (e.g., high-sensitivity troponin) was limited or unavailable in our study sample. Thus, we cannot completely rule out the potential impact of these variables on our study endpoints. Third, the SAPS II score was only available as a whole and not as its separate components. Since it was obtained within the first 24 h after ICU admission, it may not always reflect the severity of illness before ICU admission. In addition, SAPS II as an estimate of case severity and ICU mortality is not a specific marker for neurological impairment as the National Institutes of Health Stroke Scale (NIHSS) and does not thoroughly reflect the neurologic deficits upon ICU admission [53]. Fourth, the NEMS score was originally designed to measure the burden of nursing care. Thus, interventions like mechanical ventilation, renal replacement therapy, use of vasoactive agents are only surrogates for patients’ acuity. Fifth, our dataset does not provide information regarding the burden of cardiovascular risk factors and their change over time. However, it seems questionable to what extent these factors play a key role in our investigation since stroke and AMI show different temporal trends. Indeed, as both conditions are associated with the same risk factors, one would expect that their modification results in similar outcomes. Finally, our study was conducted in stroke patients admitted to the ICU and did not include stroke unit admissions, thereby assuming more severe illness in our cohort as compared to intermediate care or stroke unit populations. Consequently, our data may not be extrapolated to patients admitted to stroke units and are limited to the most critically ill.

## Conclusion

Our data showing time trends over 12 years in younger individuals with AMI or stroke suggest that gender differences in ICU admission, case severity and mortality still exist in patients with AMI, while the gender gap in the provision of care is closing in stroke patients, resulting in improved outcomes of critically ill women. Although our data are limited by potential confounders not available from the registry, our study emphasizes that triage and selection criteria for ICU admission and invasive treatments, particularly in younger individuals with AMI, should be carefully reassessed. Further research is needed to identify sex- and gender-specific disease modifiers during ICU stay in this population.

## Supplementary Information

**Additional file 1**: **Table S1**. STROBE Statement—Checklist of items

## Data Availability

Data are available from the University Hospital Basel Institutional Data Access for researchers who meet the criteria for access to confidential data.

## Abbreviations

AMI: Acute myocardial infarction
EKNZ: Ethics Committee of Northwestern Switzerland
FSO: Swiss Federal Statistical Office
hs-cTnT: High-sensitive troponin
ICU: Intensive care unit
MDSi: Minimal Dataset for ICUs
SAPS II: Simplified Acute Physiology Score II
SSICM: Swiss Society of Intensive Care Medicine
NEMS: Nine Equivalents of Nursing Manpower Use Score
NIHSS: National Institutes of Health Stroke Scale
